# Study protocol: ACCESS: (Advancing Contraceptive Equity and Service Uptake through Telemedicine in the US Safety-Net)

**DOI:** 10.21203/rs.3.rs-6256504/v1

**Published:** 2025-08-25

**Authors:** Blair G. Darney, Brenda M. McGrath, Shelby L. Watkins, Annie E. Larson, Laura D. Lindberg, Erika K. Cottrell

**Affiliations:** Oregon Health and Science University; OCHIN, Inc. Portland; OCHIN, Inc. Portland; OCHIN, Inc. Portland; Rutgers School of Public Health; OCHIN, Inc. Portland

**Keywords:** Telemedicine, contraceptive care, women’s health, reproductive health, electronic health records, community health centers

## Abstract

**Background:**

Access to desired contraceptive care is a critical component of reproductive autonomy. Telemedicine (TM), or the remote provision of clinical services via technology, in community-based health centers has the potential to expand access to family planning services, potentially enhancing both reproductive autonomy and equity. However, little is known about which patient populations use TM for contraceptive services in the US “ safety net” (community-based health centers), if there are inequities in access to TM care, or patient preferences for TM contraceptive care. Also unknown are potential unintended consequences may result from using TM versus face-to-face visits for contraceptive care.

**Methods:**

This paper describes the protocol for a 5-year, multilevel, mixed-methods study examining the use of TM for contraceptive services across a large network of over 2400 US community-based health centers serving millions of patients. Quantitative analyses will use electronic health record data from the ADVANCE network to identify differences in the use of TM for contraceptive services and quantify inequities or unintended consequences of such use for individuals and the health system. Quantitative outcomes include the use of TM versus face-to-face visits for any contraceptive care, contraceptive method switching, no-show and cancellation rates, and access to long-acting reversible contraception (LARC). Quantitative analyses will include variables at the patient, clinic, and contextual (census tract of patient address and state of clinic location) levels. The qualitative investigation will focus on experiences with TM and factors that may impact access to contraceptive services through TM versus in-person care, providing a comprehensive understanding of both statistical trends and underlying contextual dynamics.

**Discussion:**

Our study will provide real-world evidence about use of TM for contraceptive services in the US “safety net”. Our results will help us understand the potential for TM to expand access to contraceptive care and any unintended consequences. Our findings will have broad implications for reducing disparities in contraceptive care access and can inform best practices for TM delivery, as well as policy decisions about payer reimbursements for different TM strategies for contraceptive services.

## BACKGROUND

Access to desired contraceptive care is a component of people’s reproductive autonomy(1), or the ability to be fully empowered in reproductive decisions and to access reproductive health services without interference or coercion(1, 2). Telemedicine (TM) has the potential to expand access to effective contraception in a format that may be more accessible and acceptable to patients. Moreover, use of TM to expand access to contraceptive services to those facing barriers to care offers the potential to increase sexual and reproductive health equity(3). TM expanded rapidly nationwide during the COVID-19 pandemic(4) and could continue improving access to contraceptive care(5, 6). Widespread use of TM for contraceptive care was limited prior to the COVID-19 pandemic, partly due to restrictions for reimbursement and costs of implementation(7); many of these barriers were overcome during the COVID-19 pandemic, but may currently be sunsetting, making this an opportune time to examine use of TM for contraceptive services. TM has been identified a priority area for health policy–focused contraceptive research(8).

Telemedicine has been shown to be safe and effective in delivery of reproductive health services(9–11) including hormonal contraceptive methods. Building on this evidence, prior research focused on TM for contraceptive services in a commercially insured population prior to the COVID-19 pandemic revealed that the top diagnoses for reproductive health visits delivered via TM were for counseling and advice on contraception and for surveillance for oral contraceptives(12). Among a small sample (n = 172) of family planning clinicians in the US during the early months of the COVID-19 pandemic, experience with TM was positive and clinicians felt TM should be expanded(10). Similarly, evidence suggests patients are satisfied with TM for contraceptive services(10, 13, 14).

Despite its promise, inequities in access to TM for contraceptive services may still exist. Online platforms providing oral contraception and counseling do not accept Medicaid insurance in all states, and out-of-pocket costs vary widely. Evidence on TM utilization patterns is mixed. For example, one study found that higher-income individuals are less likely to choose TM, while those with public insurance may be more likely than those with commercial insurance to use it (Meurice). However, another study found that the uninsured are either less likely (Yarger) or more likely (Lindenberg) to use TM(15–17).

The publicly funded family planning “safety net” system of community-based health centers - including Federally-Qualified Health Centers (FQHCs) and look-alikes, and Title X clinics - is a critical provider of family planning services to historically marginalized populations. Moving forward, we will use the term community-based health centers to describe this grouping. However, existing evidence about contraceptive services across community-based health centers is limited by fragmentation of care, programs and payors, as well as by small samples, clinic or program-level analysis, and/or the exclusion of the uninsured from research(18–23). It is not known which patient populations use TM for contraceptive services across US community-based health centers, if there are inequities in access to TM, if there are unintended consequences such as unwarranted differences in method type, access to method switching, or differential follow-up by visit modality(10). Furthermore, a recent systematic review of TM for women’s preventive services(11) identified key evidence gaps around access to care, health equity, and potential harms or unintended consequences of TM.

This protocol paper describes a mixed-methods study designed to address these gaps. Using real-world electronic health record (EHR) data, we will examine patterns of TM use for contraceptive services across a national network of community-based health centers. We will quantify inequities in access and utilization, identify any unintended consequences of TM utilization, and explore patient experiences and preferences for TM in contraceptive care.

### Specific Aims

#### Aim 1.

Assess whether there is a differential uptake of the use of TM for contraceptive services across community-based health centers. We will focus on key populations (e.g., adolescents, uninsured, Latinas, Black women, rural), clinic characteristics (e.g., Title X vs non-Title X clinics), community-level social determinants of health (SDH) factors (e.g., social and economic conditions, computer and broadband access), and state-level policy factors (e.g., Medicaid expansion) to identify differential uptake in the use of TM (versus face-to-face visits) for contraceptive services for low-income populations.

#### Aim 2.

Quantify inequities or unintended consequences of TM utilization versus face-to-face visits for individuals and the health system. These analyses will focus on women with evidence of a visit for contraceptive care. The following outcomes will be examined: 1) access and utilization of long-acting reversible contraception (LARC), which requires an in-person visit for placement; 2) rates of method switching at one year; and 3) other unintended consequences such as no-show or cancellation rates. We will identify any disparities in these patterns by individual, health center, community, or state-level factors as in Aim 1.

#### Aim 3.

Understand patient experience of care and preferences for contraceptive services via TM versus in-person. We will conduct semi-structured interviews with patients who receive contraceptive care at community-based health centers to understand their experiences with TM, barriers and facilitators to receiving contraceptive services through TM versus in-person care, and contextual factors that impact their ability to access contraception through TM versus in-person care.

## METHODS

### Setting

This study will use real-world EHR data from clinics that are part of the Accelerating Data Value across a National Community Health Center Network (ADVANCE) Clinical Research Network (CRN). ADVANCE is led by OCHIN in partnership with Health Choice Network (HCN), Fenway Health, University of Washington, and Oregon Health & Science University. ADVANCE is one of eight PCORnet CRNs funded by PCORI and the ADVANCE Data Warehouse includes research-ready EHR data, formatted in the PCORnet common data model. The ADVANCE Data Warehouse contains clinical data for the 13.7 million distinct patients who received care across 269 community-based health centers and more than 2,400 active clinical sites in 38 states. This is one of the nation’s most comprehensive health datasets on patient care and outcomes for uninsured, publicly insured, and historically underserved populations.

### Quantitative Methods and Analysis

#### Sample.

We will utilize EHR data from the ADVANCE Data Warehouse to assess differences in the uptake of TM contraceptive services (Aim 1) and identify inequities or unintended consequences of TM utilization (Aim 2). Our sample will include patients who are documented in the EHR as 1) female, 2) at risk for pregnancy, 3) of reproductive age (i.e., 15–49 years old), and 4) received contraceptive care in an ADVANCE community-based health centers between January 1, 2019, and December 31, 2027. Patients will be determined to be at risk for pregnancy in the absence of any EHR documentation of permanent contraception, infecundity, or current pregnancy. Visits only for the provision of non-prescription methods (e.g., condoms and spermicide) will be excluded as this data is not well captured in the clinical EHR data. Permanent contraception is not provided at community-based health centers, we therefore do not include permanent contraception in this study. Contraceptive care is defined as receipt of contraceptive services or procedures by ICD or CPT code (Appendix), or by prescription of contraceptives (oral contraceptive pills, injections, patches, rings, a long-acting reversible contraceptive (LARC) method (intrauterine device or implant), or emergency contraception)(24).

#### Dependent Variables.

Dependent variables include contraceptive care visit modality (TM or face-to-face); LARC placement; method satisfaction, as measured by rate of method switching at one year); and unintended consequences, including appointment no-shows or cancellations following a contraceptive counseling visit. TM refers to billable synchronous communication between a patient and provider, such as through audio-only or video and audio, as well as through asynchronous virtual communication, including electronic visits (eVisits) or electronic consultations (eConsults)(25).

#### Independent variables.

We will include additional covariates at the *individual, clinic, community*, and *state levels* (Table 1). At the individual patient level, we will include demographic and Social Determinants of Health variables such as age, percent FPL, type of insurance used (including uninsured), urban/suburban/rural status, race/ethnicity, migrant worker status, and preferred language. At the clinic level we will include clinic Title X status, and patient (sex/age and race/ethnicity) and provider (women’s health specialists) mix. At the community/ ZCTA level, we will include community-level SDH variables such as percent with broadband service, percent with a computer in the home, and percent that use smartphones to access the internet (from the American Community Survey, ACS), which could all impact ability to access care via TM. We will explore additional community-level SDH factors at the ZCTA-level that are integrated into the ADVANCE CRN, including percent with a college degree, racial/ethnic segregation, immigrant integration, and social deprivation index (SDI) percentile. At the state level, following our previous research, we will include Medicaid expansion, Medicaid eligibility threshold, and state family planning program (1115 waiver or SPA).

#### Statistical Analysis.

We will use generalized linear mixed models (GLMMs). Models will incorporate random effects for clinics to account for the nested structure of the data, with patients nested within clinics, and account for both within-group correlation and between-group variability. To ensure the validity of any random effects included in the models, we will use the Hausman test(26). All independent variables at the individual, clinic, community, and state level, as well as time, will be fixed effects in the models.

First, we will conduct a descriptive analysis of the data to provide an overview of the key characteristics and trends. We will summarize the distribution of patient demographics, clinic-level factors, state-level factors, and the utilization of TM versus face-to-face visits for contraceptive care.

Next, using logistic mixed-effects regression at the visit level, we will incorporate individual-, clinic-, and state-level factors as fixed effects to explore their associations with TM utilization (Aim 1). Clinic random effects will be included to account for the nested structure of the data, with patient visits nested within clinics. The binary outcome for the model will be the use of TM versus face-to-face for a contraceptive visit among women of reproductive age who received contraceptive care.

Additionally, we will investigate whether differences in TM uptake for contraceptive services exist among specific patient populations, such as adolescents, uninsured patients, and Black and Latina women. The goal of this aim is to identify multilevel factors associated with TM use for contraceptive services and generate a better understanding of inequities in TM utilization. To complement the global model, we will conduct stratified analysis by age (adolescents vs. older individuals), visit type (counseling vs. provision), and method type (moderately vs. most effective). See Table 1 for variable definitions.

In Aim 2, the following outcomes will be examined: 1) long-acting reversible contraception (LARC), which requires an in-person visit for placement (visit level); 2) rates of method switching at one year as a proxy of method satisfaction (woman level); and 3) other unintended consequences including no-shows or cancellations following a contraceptive counseling visit (woman level). For the first model, initiation of a LARC method following counseling will be the outcome and visit modality (TM versus face-to-face) will be the key independent variable. For this model, we will compare most effective vs. moderately effective methods. This will allow us to assess whether the odds of LARC initiation differ by counseling modality after adjusting for patient, clinic, and state-level factors (Table 1). We will also consider interactions between modality and key factors that might make in- person care more difficult to obtain, such as adolescent age and rural residence.

To examine contraceptive method switching by visit modality, we will use a logistic mixed-effects model. We will adjust for patient- and clinic-level factors and consider effect modification by conducting subgroup analyses based on age, race/ethnicity, insurance type, and clinic type. Additional analyses will investigate the patterns of method switching (i.e. transitioning from pill to LARC or vice versa) and identify if TM or in-person visits are differentially associated with these patterns.

To control for selection bias in who utilizes TM and isolate the causal effect of TM on LARC initiation and method switching, we will employ propensity score weighting using inverse probability of treatment weighting (IPTW). IPTW is a statistical technique that balances the observed covariates between the TM and face-to-face groups, thus reducing the potential bias introduced by non-random treatment assignment. First, we will estimate the propensity scores for TM utilization by fitting a logistic regression model that includes patient demographics, clinic-level factors, state-level factors, and other relevant covariates. Next, we will use the inverse of the propensity scores to compute stabilized weights for each individual. These weights will be applied in the logistic mixed-effects models for LARC initiation and method switching to account for the differential selection probabilities between TM and face-to-face groups. We will assess the balance of covariates between the TM and face-to-face groups after applying the propensity score weights to ensure that the weighting method effectively reduces the bias in observed covariates between the two groups. We will then estimate the causal effect of TM utilization on LARC initiation and on method switching, adjusting for potential confounding factors.

To examine whether TM LARC counseling is associated with an increase in no-show/cancellation rates for subsequent in-person visits (which are assumed to indicate the intention for LARC placement) relative to baseline levels among women receiving contraceptive care, we will conduct a GLMM. This analysis will include all women who had any contraceptive care visit during the study period. For each patient, we will identify whether the in-person appointment was scheduled within 30 days following a LARC counseling session as the primary independent variable of interest. The primary outcome will be the appointment no-show or cancellation status, defined as either a no-show or canceled appointment versus an attended appointment. Additionally, we will conduct a separate analysis for in-person appointments scheduled within 60 days following LARC counseling to assess whether the effect of TM counseling on no-show rates varies depending on the timing of the follow-up visit. We will adjust for patient-level covariates, such as age, socioeconomic status, race/ethnicity, and insurance status, as well as clinic-level covariates, such as clinic type, while accounting for the nested nature of patients within clinics.

### Qualitative Methods and Analysis

#### Sample.

We will recruit up to 4 OCHIN community-based health centers to take part in the qualitative portions of the study. Informed by the quantitative findings in Aim 1, we will purposely recruit community-based health centers from different geographic regions with varying levels of telemedicine implementation. At each healthcare organization we will attempt to recruit 9–10 patients; sampling will be informed by quantitative results to focus on patient populations using and less likely to use TM. We aim to conduct up to 40 patient interviews.

#### Data Collection.

We will coordinate recruitment efforts and conduct semi-structured interviews with patients in each site. The study team will work with clinic staff to identify potential participants through active recruitment (e.g., through outreach from a clinic staff member) and/or sending out a letter about the study to a list of potential participants with a number to call for more information. Interviews will be conducted by phone and/or secure video conference (i.e., Zoom) and will last 45–60 minutes. Patients will receive a $60 gift card for their time. The goal of the semi-structured interviews will be to elicit patients’ experiences with receiving contraceptive care in general, including barriers and facilitators to accessing contraception; their perspectives on telemedicine versus in-person care, barriers and facilitators to accessing or utilizing telemedicine for contraception, and any contextual factors that many have impacted their experiences (e.g. access to technology, work schedule, insurance access, finding a private place to have their telemedicine visit). Participating healthcare organizations will have the opportunity to review and provide input on our interview guide and the guide will be iteratively adapted based on the initial set of 4–5 interviews.

#### Data Analysis.

All interviews will be professionally transcribed and entered into a qualitative analysis software. We will follow the 5-phase analysis strategy as described by Crabtree and Miller(27). Describing: A process of reading the data (immersion) to identify overarching themes for organization (crystallization). Data will be discussed during regular team meetings to identify theme and variation. Organizing: Developing a system for organizing the data to structure the subsequent analysis by reviewing themes and creating a coding template. Connecting: The team will review the coded text by theme, making connections between issues and comparing perceptions between the different clinics as well as between the different states. Corroborating/Legitimizing: We will seek out additional data to confirm, disconfirm, or refute insights and seek additional clarification from interviews or other data sources as needed. Because qualitative data collection will be ongoing and concurrent with quantitative analyses, we can adapt our quantitative analysis to explore unique findings that emerge from the qualitative data. Conversely, we can adapt our interview guide to further explore unexpected findings from our quantitative analyses. Representing: We will prepare detailed descriptions of the themes and variation arising from the data, illustrated with audio and video clips from patient interviews.

### Limitations

#### Limitations of EHR Data.

EHR data are not collected for research purposes but are more reliable than subjective self-reported data and contain much more detailed information than claims data. Our research team has successfully conducted multiple validation studies(28–32); these proven methods can be replicated to reduce any limitations there will be with using EHR data. Nonetheless, there is a risk of misclassification—for example, inaccuracies in coding visit modality may occur(33), which could bias estimates of telemedicine uptake. Despite our extensive experience in EHR data validation and our rigorous methods to minimize coding errors, some degree of misclassification is inevitable.

While we use propensity score weighting (IPTW) to address selection bias, unmeasured confounders— such as patient preferences, provider practices, and local policy changes—may still influence outcomes. To help address this, we complement our quantitative analysis with qualitative interviews exploring patient experiences, preferences, and contextual factors that are difficult to capture in EHR data. While qualitative findings cannot eliminate unmeasured confounding, they provide valuable insights into mechanisms shaping telemedicine use and contraceptive care.

Additionally, the study period includes significant policy shifts, such as changes in telemedicine reimbursement and Medicaid continuous enrollment following COVID-19. These shifts may have unevenly impacted TM uptake(34), complicating trend interpretation and limiting the external validity as policies and clinical practices continue to evolve. We will account for state-level policy differences in our analyses.

#### Patients seeking care outside of ADVANCE networks.

Patients may receive contraceptive care outside the ADVANCE system—for example, in private practices or other health systems—particularly for follow-up visits, LARC placements, or method switching. This limitation may lead to an underestimation of overall service utilization and outcome rates. Moreover, patients who seek care outside the network could differ systematically from those who remain within it, thereby introducing selection bias and affecting the generalizability of our findings to the broader population.

#### Limitations of qualitative data.

Qualitative interviews allow us to gain a valuable window into the day-to-day experiences and perceptions of people who receive contraceptive care in community-based health centers, to understand the deeper meaning and context that underlie patterns arising in the quantitative data, and to identify potential future areas for quantitative exploration. Due to time and resource constraints, we will recruit patients from four community-based health centers within the OCHIN network. Although we will attempt to recruit a broad sample of patients, we will be unable to capture all possible perspectives or experiences. Despite these known limitations, findings from our qualitative analysis will provide important insights that help to contextualize our quantitative findings by illuminating patient experiences, preferences, and perspectives on contraception access via both in-person and TM visits in community-based health centers.

## STUDY STATUS

This protocol is based on a proposal reviewed and funded by the National Institute on Minority Health and Health Disparities in 2024(35). At the time of submission, the research team is preparing for our first quantitative analysis.

## DISCUSSION

Although the recent expansion of TM in primary care, including in the ADVANCE network of community health centers, in response to the COVID-19 pandemic has the potential to improve access to contraception services via TM among low-income and vulnerable populations, there is a growing need to capture the opportunities but also any potential negative consequences or harms of the scale-up and widespread adoption of TM for contraceptive services. Currently, much of the TM literature focuses on expanding access, but emerging evidence in small samples has also documented challenges to using TM, concerns about widening inequities in access to care, racial and social determinants of health (SDH) inequities in the use of TM, and suggests some patient populations prefer in-person care(10)(36, 37). Moreover, this study will consider patient preferences and perspectives on the use of TM within a low-income and diverse population, a critical and understudied component of TM implementation. Also, due to our unique data source we will be able to assess practice and policy changes nearly in real-time; our study period includes, for example, the end of the public health emergency and “unwinding” of Medicaid continuous enrollment, and possible sunsetting of COVID-era TM policies.

## CONCLUSION

Our findings will be relevant in informing practice by providing evidence around patterns of TM use in a large network of community-based health centers and identifying any inequities in access or unintended consequences. Qualitative findings about patient perspectives on using TM for contraceptive services will enrich quantitative findings. Together, our findings can inform the design implementation of TM and decisions about payer reimbursement for different TM strategies.

## Supplementary Files

This is a list of supplementary files associated with this preprint. Click to download.

- AppendixProtocolPaper20250317.docx

## Figures and Tables

**Table 1:** Description of independent variables, ACCESS

Variable	Description
Time	Month of service for descriptive time trend analyses (Aim 1); Quarter or year for regression analyses (Aim 2)
**Visit-level Factors**	
Contraceptive care type	Counseling, surveillance, or service provision (initiation and discontinuation)
Method type	Individual methods, grouped into most (LARC) and moderately (hormonal) effective, per OPA definitions
**Individual-level Factors**	
Age	5-year age categories, adolescent vs. adult
Federal Poverty Level (FPL)	<100%, 100–149%, 150–199, ≥ 200% FPL
Insurance used	Public, private, uninsured
Urban/suburban/rural status	Based on USDA Rural-Urban Commuting Area Codes82
Race/Ethnicity	Latina, non-Latina white, non-Latina Black, non-Latina Asian, Other
Migrant worker status	Yes, no
Preferred language	English, Spanish, other
**Clinic-level Factors**	
Patient mix: ethnicity/Race	Percent of patients identified as Latina; Percent of patients identified as Black
Patient mix: sex and age	Percent of patients who are women of reproductive age (15–49 years)
Provider mix	Women’s health specialists on staff
Title X status	Clinic participates in federal Title X program
**Community-level (census tract-level) Factors**	
Broadband access	Percent of households with broadband access (calculated from American Community Survey (ACS))
Computer access	Percent of households with desktop or laptop (ACS)
Smartphone use	Percent of households using smartphones to access internet (ACS)
Race/ethnic residential segregation (Dissimilarity Index, Separation Index)	Measure of neighborhood racial/ethnic residential segregation (ACS)
Educational Attainment	Percent of people with a college degree (ACS)
Immigrant integration	Percent Latinx; Percent with difficulty speaking English (all ACS)
Social vulnerability index	Census tract level social vulnerability index^a^ percentile score developed by the CDC using ACS data
Poverty	Percent of residents with household incomes < 100% FPL (ACS)
**State-level Health Policy Factors**	
Medicaid expansion status	Expansion vs. non-expansion states
Medicaid threshold	State Medicaid eligibility threshold^b^
Family planning program	State Family Planning Program: 1115 waiver or SPA
TM reimbursement	National Conference of State Legislatures. State Telehealth Policies

a. CDC. Social Vulnerability Index [Internet]. Place and Health - Geospatial Research, Analysis, and Services Program (GRASP). 2024. Available from: https://www.atsdr.cdc.gov/place-health/php/svi/?CDC_AAref_Val=https://www.atsdr.cdc.gov/placeandhealth/svi/index.html

b. KFF. Medicaid Income Eligibility Limits for Adults as a Percent of the Federal Poverty Level [Internet]. KFF. 2024. Available from: https://www.kff.org/affordable-care-act/state-indicator/medicaid-income-eligibility-limits-for-adults-as-a-percent-of-the-federal-poverty-level/?currentTimeframe=0&sortModel=%7B%22colId%22:%22Location%22

## Data Availability

The datasets generated and analyzed in this study are not publicly available because they are from OCHIN Epic, a privately hosted electronic health record system. Datasets are available through the corresponding author on reasonable request.

## Abbreviations

TM: Telemedicine
ADVANCE: Accelerating Data Value Across a National Community Health Center Network
LARC: Long-acting reversible contraception
SDH: Social determinants of health
CRN: Clinical Research Network
HCN: Health Choice Network
ICD: International Classification of Diseases
CPT: Current Procedural Terminology
GLMM: Generalized linear mixed models
IPTW: Inverse probability of treatment weighting

## References

[1] Fuentes-Afflick E, Perrin JM, Moley KH, Díaz Á, McCormick MC, Lu MC. Optimizing Health And Well-Being For Women And Children: Commentary highlights interventions and recommends key improvements in programs and policies to optimize health and well-being among women and children in the United States. Health Affairs. 2021;40(2):212–8. 33476200 10.1377/hlthaff.2020.01504

[2] Senderowicz L, Higgins J. Reproductive autonomy is nonnegotiable, even in the time of COVID-19. International Perspectives on Sexual and Reproductive Health. 2020;46:147–51. 32790638 10.1363/intsexrephea.46.2020.0147

[3] Hart J, Crear-Perry J, Stern L. US sexual and reproductive health policy: which frameworks are needed now, and next steps forward. American Journal of Public Health. 2022;112(S5):S518–S22. 35767777 10.2105/AJPH.2022.306929 PMC10490305

[4] Temesgen ZM, DeSimone DC, Mahmood M, Libertin CR, Palraj BRV, Berbari EF, editors. Health care after the COVID-19 pandemic and the influence of telemedicine. Mayo Clinic Proceedings; 2020: Elsevier. 10.1016/j.mayocp.2020.06.052 PMC7383140 32948262

[5] Barbosa W, Zhou K, Waddell E, Myers T, Dorsey ER. Improving Access to Care: Telemedicine Across Medical Domains. Annual review of public health. 2021;42(1):463–81. 10.1146/annurev-publhealth-090519-093711 33798406

[6] Ukoha EP, Davis K, Yinger M, Butler B, Ross T, Crear-Perry J, Ensuring equitable implementation of telemedicine in perinatal care. Obstetrics & Gynecology. 2021;137(3):487–92. 33543895 10.1097/AOG.0000000000004276 PMC7884085

[7] Kane CK, Gillis K. The use of telemedicine by physicians: still the exception rather than the rule. Health affairs. 2018;37(12):1923–30. 30633670 10.1377/hlthaff.2018.05077

[8] Coalition to Expand Contraceptive Access. Priority Roadmap for Policy-Ready Contraceptive Research. Sacramento, CA; 2021.

[9] DeNicola N, Grossman D, Marko K, Sonalkar S, Butler Tobah YS, Ganju N, Telehealth Interventions to Improve Obstetric and Gynecologic Health Outcomes: A Systematic Review. Obstetrics & Gynecology. 2020;135(2). 10.1097/AOG.0000000000003646 PMC7012339 31977782

[10] Stifani BM, Smith A, Avila K, Boos EW, Ng J, Levi EE, Telemedicine for contraceptive counseling: patient experiences during the early phase of the COVID-19 pandemic in New York City. Contraception. 2021;104(3):254–61. 33861981 10.1016/j.contraception.2021.04.006 PMC8056642

[11] Cantor AG, Nelson HD, Pappas M, Atchison C, Hatch B, Huguet N, Telehealth for women’s preventive services for reproductive health and intimate partner violence: A comparative effectiveness review. Journal of general internal medicine. 2023;38(7):1735–43. 36650334 10.1007/s11606-023-08033-6 PMC9845023

[12] Weigel G FB, Ranji U, Salganicoff A. Telemedicine in Sexual and Reproductive Health kff.org: Kaiser Family Foundation; 2019 [Available from: https://www.kff.org/womens-health-policy/issue-brief/telemedicine-in-sexual-and-reproductive-health/.

[13] Freeman E, Paul R, Dorsey M, Madden T. Comparison of interpersonal quality of contraceptive counseling delivered via telehealth versus in person. Contraception. 2023;128:110129. 37499735 10.1016/j.contraception.2023.110129

[14] Shin RJ, Yao M, Akesson C, Blazel M, Mei L, Brant AR. An exploratory study comparing the quality of contraceptive counseling provided via telemedicine versus in-person visits. Contraception. 2022;112:86–92. 35247368 10.1016/j.contraception.2022.02.004

[15] Meurice ME, Mody SK, Nodora J, Marengo A, Averbach S. Social determinants of choosing telemedicine for contraceptive care: A retrospective cohort study. Contraception. 2024;134:110414. 38431258 10.1016/j.contraception.2024.110414

[16] Yarger J, Hopkins K, Elmes S, Rossetto I, Van Liefde D, De La Melena S, Use of telemedicine to obtain contraception among young adults: Inequities by health insurance. Contraception. 2024;134:110419. 38467325 10.1016/j.contraception.2024.110419 PMC11191717

[17] Lindberg LD, Mueller J, Haas M, Jones RK. Telehealth for contraceptive care during the COVID-19 pandemic: results of a 2021 national survey. American journal of public health. 2022;112(S5):S545–S54. 35767798 10.2105/AJPH.2022.306886 PMC10490317

[18] Rodriguez MI, Darney BG, Elman E, Linz R, Caughey AB, McConnell KJ. Examining quality of contraceptive services for adolescents in Oregon’s family planning program. Contraception. 2015;91(4):328–35. 25545334 10.1016/j.contraception.2014.12.008

[19] Kavanaugh ML, Zolna MR, Burke KL. Use of Health Insurance Among Clients Seeking Contraceptive Services at Title X-Funded Facilities in 2016. Perspectives on sexual and reproductive health. 2018;50(3):101–9. 29894024 10.1363/psrh.12061 PMC6135668

[20] Fowler CI, Ahrens KA, Decker E, Gable J, Wang J, Frederiksen B, Patterns and trends in contraceptive use among women attending Title X clinics and a national sample of low-income women. Contraception: X. 2019;1:100004. 32550524 10.1016/j.conx.2019.100004 PMC7286153

[21] Boudreaux M, Choi YS, Xie L, Marthey D. Medicaid Expansion at Title X Clinics. Medical Care. 2019;57(6):437–43. 30973473 10.1097/MLR.0000000000001120 PMC6522286

[22] Frost JJ, Gold RB, Frohwirth LF, Blades N. Variation in Service Delivery Practices Among Clinics Providing Publicly Funded Family Planning Services in 2010. 2012.

[23] Wood S, Goldberg DG, Beeson T, Bruen BK, Johnson K, Mead H, Health centers and family planning: Results of a nationwide study. Washington, DC: School of Public Health & Health Services, George Washington University; 2013.

[24] Darney BG, Jacob RL, Hoopes M, Rodriguez MI, Hatch B, Marino M, Evaluation of Medicaid Expansion Under the Affordable Care Act and Contraceptive Care in US Community Health Centers. JAMA network open. 2020;3(6):e206874–e. 32496568 10.1001/jamanetworkopen.2020.6874 PMC7273194

[25] Services CfMaM. Telehealth 2025 [Available from: https://www.medicaid.gov/medicaid/benefits/telehealth/index.html.

[26] Hausman JA. Specification tests in econometrics. Econometrica: Journal of the econometric society. 1978:1251–71.

[27] Crabtree B, Miller W. Using codes and code manuals: A template for organizing style of interpretation. In: Crabtree B, Miller W, editors. Doing qualitative research. 2nd Edition ed. Nuwbury Park, CA: Sage Publications; 1999. p. 163–78.

[28] Angier H, Gold R, Gallia C, Casciato A, Tillotson CJ, Marino M, Variation in outcomes of quality measurement by data source. Pediatrics. 2014;133(6):e1676–e82. 24864178 10.1542/peds.2013-4277 PMC4918742

[29] Devoe JE, Gold R, McIntire P, Puro J, Chauvie S, Gallia CA. Electronic health records vs Medicaid claims: completeness of diabetes preventive care data in community health centers. Annals of family medicine. 2011;9(4):351–8. 21747107 10.1370/afm.1279 PMC3133583

[30] Heintzman J, Bailey SR, Hoopes MJ, Le T, Gold R, O’Malley JP, Agreement of Medicaid claims and electronic health records for assessing preventive care quality among adults. Journal of the American Medical Informatics Association: JAMIA. 2014;21(4):720–4. 24508767 10.1136/amiajnl-2013-002333 PMC4078280

[31] Heintzman J, Marino M, Hoopes M, Bailey SR, Gold R, O’Malley J, Supporting health insurance expansion: do electronic health records have valid insurance verification and enrollment data? Journal of the American Medical Informatics Association: JAMIA. 2015;22(4):909–13. 25888586 10.1093/jamia/ocv033 PMC5009899

[32] Marino M, Angier H, Valenzuela S, Hoopes M, Killerby M, Blackburn B, Medicaid coverage accuracy in electronic health records. Preventive Medicine Reports. 2018;11:297–304. 30116701 10.1016/j.pmedr.2018.07.009 PMC6082971

[33] Larson AE, Stange KC, Heintzman J, Nishiike Y, McGrath BM, Davis MM, Identifying virtual care modality in electronic health record data. Learning Health Systems. 2024:e10411. 38883878 10.1002/lrh2.10411 PMC11176566

[34] Holderness H, Baron A, Hodes T, Marino M, O’Malley J, Danna M, Community Health Centers Uptake of Telemedicine During the COVID-19 Pandemic: Trends, Barriers, and Successful Strategies. Journal of Primary Care & Community Health. 2024;15:21501319241274351. 10.1177/21501319241274351 PMC11345735 39183703

[35] Health NIo. ACCESS: Advancing Contraceptive Equity and Service Uptake through Telemedicine in the US Safety-Net, 2019–2025 NIH RePORT2025 [Available from: https://reporter.nih.gov/project-details/10856800.

[36] Hill BJ, Lock L, Anderson B. Racial and ethnic differences in family planning telehealth use during the onset of the COVID-19 response in Arkansas, Kansas, Missouri, and Oklahoma. Contraception. 2021;104(3):262–4. 34058223 10.1016/j.contraception.2021.05.016

[37] Yarger J, Hopkins K, Elmes S, Rossetto I, De La Melena S, McCulloch CE, Perceived access to contraception via telemedicine among young adults: inequities by food and housing insecurity. Journal of general internal medicine. 2023;38(2):302–8. 35657468 10.1007/s11606-022-07669-0 PMC9165539

[38] Alvidrez J, Castille D, Laude-Sharp M, Rosario A, Tabor D. The national institute on minority health and health disparities research framework. American journal of public health. 2019;109(S1):S16–S20. 30699025 10.2105/AJPH.2018.304883 PMC6356129

